# Current Concept in Adult Peripheral Nerve and Brachial Plexus Surgery

**DOI:** 10.1055/s-0037-1606841

**Published:** 2017-10-02

**Authors:** Lukas Rasulic

**Affiliations:** 1 School of Medicine, University of Belgrade, Belgrade, Serbia; 2 Clinic for Neurosurgery, Clinical Center of Serbia, Belgrade, Serbia

**Keywords:** endoscopy, nerve repair, reinnervation

## Abstract

Peripheral nerve injuries and brachial plexus injuries are relatively frequent. Significance of these injuries lies in the fact that the majority of patients with these types of injuries constitute working population. Since these injuries may create disability, they present substantial socioeconomic problem nowadays. This article will present current state-of-the-art achievements of minimal invasive brachial plexus and peripheral nerve surgery. It is considered that the age of the patient, the mechanism of the injury, and the associated vascular and soft-tissue injuries are factors that primarily influence the extent of recovery of the injured nerve. The majority of patients are treated using classical open surgical approach. However, new minimally invasive open and endoscopic approaches are being developed in recent years—endoscopic carpal and cubital tunnel release, targeted minimally invasive approaches in brachial plexus surgery, endoscopic single-incision sural nerve harvesting, and there were even attempts to perform endoscopic brachial plexus surgery. The use of the commercially available nerve conduits for bridging short nerve gap has shown promising results. Multidisciplinary approach individually designed for every patient is of the utmost importance for the successful treatment of these injuries. In the future, integration of biology and nanotechnology may fabricate a new generation of nerve conduits that will allow nerve regeneration over longer nerve gaps and start new chapter in peripheral nerve surgery.

## Introduction

History of peripheral nerve surgery dates back to the year 1608 when the first reconstruction of transected nerve was performed by Ferara. Modern peripheral nerve surgery started in 1964 when Curtze started using operative microscope. Development of high-tech equipment and materials made possible for peripheral nerve surgery to grow; consequently, nowadays, its possibilities are exponentially improved. Precise diagnostics, state-of-the-art microsurgical technique, and minimally invasive approaches made huge improvement in treatment outcome. Cooperation of neurosurgeon; orthopaedic, vascular, and plastic surgeon; physiatrist; physiologist; neurologist; and radiologist is essential in the treatment of peripheral nerve injuries. The aim of this article is to present current accomplishments and limitations of peripheral nerve and brachial plexus surgery, analyzing available literature.

Peripheral nerve injury is relatively common and occurs primarily from trauma or sometimes as a complication of surgery. Traumatic injuries can occur due to stretch, crush, laceration, and ischemia and are more frequent in times of war. It is considered that approximately 5% of all trauma patients have peripheral nerve or brachial plexus injury (BPI). 1

### Pathophysiology of the Peripheral Nerve Injury

Following a nerve injury, the axons undergo degenerative processes, and subsequently they attempt regeneration. Almost immediately after injury, Wallerian degeneration begins, sealing the severed axon ends and initiating the regenerative phase. 2 After this, decreased production of neurotransmitters and increased production of materials necessary for regeneration begin. 3

Over the first few days following peripheral nerve injury, the axons in the distal nerve stump will degenerate. However, the myelin sheath and the basal lamina provided by the Schwann cells remain intact. 4 The presence of macrophages at the site of injury stimulates the proliferation of Schwann cells in the distal stump. 5 6 The proliferation of Schwann cells within their basal lamina leads to the formation of tube-like structures, Bands of Büngner, which provide a guide so that axons regenerating from the proximal stump can reach their targets. 7 Spontaneous functional recovery is dependent on the number of correctly matched motor and sensory neurons.

### Patient Evaluation

The first step in the adequate evaluation of every patient is obtaining detailed patient history. Next, a thorough neurological and clinical examination must be performed. After these two essential segments of patient assessment, electrophysiological evaluation and sometimes neuroradiological examination, magnetic resonance imaging (MRI), computed tomography (CT), and high-resolution ultrasonography are performed. Electromyoneurography performed 2 or 3 weeks after injury shows fibrillations and, later on, denervation potentials. MRI, CT, and ultrasonography are adjuvant methods that can show partial or complete transection of the nerve or compression between bone fragments. 8

Primary factors that influence the extent of recovery of the injured nerve are the age of the patient, the mechanism of the injury, and the associated vascular and soft-tissue injuries. 9

In a first-degree injury, according to Sunderland classification, patient history usually includes a blunt injury (stretch or compression). In this situation, the nerve continuity is intact as well as all the layers of the connective tissue. As a result, there is no presence of Tinel's sign at the site of injury. With this degree of injury, management is conservative and full recovery is expected.

Second- and third-degree injuries according to Sunderland classification are clinically differentiated from first-degree injuries because Tinel's sign will develop and then advance as the axons regenerate. These injuries are also managed conservatively. Full recovery is expected after a second-degree injury.

Surgical intervention is indicated with fourth- and fifth-degree injuries. In practice, any open wounds in which nerve injury is suspected should be explored, while closed injuries are usually followed up expectantly with investigative techniques such as electromyography or nerve-conduction studies. If nerve function does not recover after the initial 3-month period after the injury, surgical exploration is performed. 8

Electrophysiological assessment with nerve conduction studies and needle electromyography are useful in the evaluation of closed injuries that have not recovered within the first 3 months following the injury. The electrophysiological parameters such as conduction slowing, blocking, or failure evaluate the gross dysfunction of the peripheral nerve.

However, electrophysiological assessments can falsely localize focal lesions because the proximal parts of the peripheral nerve are typically not amenable to electrophysiological evaluation. In these situations, MRI is increasingly used as it has high specificity and sensitivity when evaluating focal injuries such as cervical nerve root avulsions or other BPIs. 10

Considering all the above, clear indications for surgical treatment are: 8

- Open injuries with apparent transection of the nerve continuity.
- Closed injuries that show no signs of recovery 3 months after injury.
- Progressive neurological deficit because of the scaring or vascular compression.
- Pharmacoresistant chronic neurogenic pain, even if neurological recovery after surgery is not to be expected.

### Open Surgical Treatment

Over the past years, surgical techniques have improved tremendously for any nerve repair, and an understanding of the nerve topography will enable the surgeon to align the motor and/or sensory fascicles correctly. This will ensure good nerve regeneration and also optimize functional recovery. During nerve repair, it is important to consider the longitudinal extent of the injury. The nerve ends should be resected sufficiently to reveal the normal fascicular pattern.

As we know, there are four main types of surgical treatment of peripheral nerve injury: (1) neurolysis; (2) end-to-end suture; (3) nerve grafting; and (4) nerve transfer.

*Neurolysis* can be performed as the only surgical procedure with lesions in continuity, or it can be performed during preparation of the nerve stumps for suture.

*Primary end-to-end neurorrhaphy* is the most desirable approach for reparation of peripheral nerve injuries when the gap between the two ends of the nerve is relatively short. 11

Following complete transection of a nerve, the nerve ends will retract, due to their elasticity. When this occurs, it is impossible to perform direct end-to-end suture.

In contaminated wounds, primary repair should not be undertaken; however, nerve ends should be approximated and marked using colored stitches during initial debridement to prevent the retraction and to ease dissection of the nerve stumps in the course of second surgery. 12

In the case of greater defects or longer gaps between the cut ends, neurorrhaphy will cause excessive tension at the repair site that will impair microvascular flow in the nerve tissue and lead to excessive scarring at the repair site. 13 In these situations, primary neurorrhaphy should not be performed, and a suitable alternative should be considered. 9

*Nerve grafting* is usually performed when nerve tissue defect is longer than 2 cm, after all the additional procedures for approximation of the nerve stumps without tension.

There are several types of grafting:

- Cable grafting.
- Interfascicular grafting.
- Fascicular grafting.
- Vascularized grafting.

Advantages of interfascicular nerve grafting are better approximation of nerve and graft diameter, better orientation of the fascicles, thin graft that gets nutrients by diffusion from its bed, better graft revascularization, and less scarring. However, there are also limitations of nerve grafting—two suture margins that are potential obstacle to the axon growth, difficulty in the identification of the appropriate fascicular groups in longer defects, and scaring of the distal suture margin or graft itself in longer defects.

There has been a significant amount of research dedicated to the development of synthetic nerve conduits for short nerve gaps that are not amenable to primary tensionless end-to-end neurorrhaphy. Using nerve conduits, donor-site morbidity, such as pain, scarring, neuroma formation, and permanent loss of sensation of the area supplied by the donor nerve, is prevented. 14 Currently, several commercially available synthetic nerve conduits have been approved by the U.S. Food and Drug Administration for peripheral nerve repair and include collagen, degradable biological material derived from bovine Achilles tendon or a combination of polyglycolic acid and polylactide caprolactone, both of which are degradable synthetic aliphatic polyesters. A majority of published studies are showing that outcome of recovery is similar as when using autograft. 15 16 17

*Nerve transfer (neurotization)* involves repair of a distal denervated nerve element using different proximal nerve as the donor of neurons and their axons to reinnervate the distal targets. The concept is to sacrifice the function of a lesser-valued donor muscle to revive function in the recipient nerve and muscle that will undergo reinnervation. Nerve transfer procedures are increasingly performed for repair of severe BPI, in which the proximal spinal nerve roots have been avulsed from the spinal cord.

Functional priorities in nerve transfer of adult BPIs are (in the following order):

1. Elbow flexion.
2. Shoulder stabilization.
3. Abduction and external rotation of the shoulder.
4. Sensory function of the thumb and index finger.
5. Hand function.

Despite advancements in the precision of microsurgical techniques, full functional recovery following peripheral nerve repair cannot always be achieved. Primary tensionless end-to-end repair should be performed whenever possible. For longer nerve gaps, the use of autologous nerve grafts is the current “gold standard.” Over the past few years, the use of the commercially available nerve conduits for bridging short nerve gap has increased. The evolution of tissue engineering and the use of biodegradable conduits for reconstruction of nerve gaps have shown promising results. 8

## Surgical Treatment

During the past few years, technological development led to creation of new, minimally invasive surgical techniques, growing in every part of surgery, and it found its place in peripheral nerve and brachial plexus surgery.

### Minimally Invasive Open Peripheral Nerve and Brachial Plexus Surgery

In the past 10 years, experts in peripheral nerve surgery all over the world are developing more and more minimally invasive open peripheral nerve surgery techniques. Besides outstanding knowledge of anatomy, diagnostic procedures had to be more precise enough to correctly evaluate injury that occurred in particular patient. Diagnostics had to be able to localize injury, give information about degree and type of injury, and in some cases visualize injury and its position in reference to other nearby structures. With this improved knowledge, minimal skin incision can be made and with it, there is minimal additional injury of surrounding tissue (especially vascular elements) thus providing optimal conditions for neuronal regeneration.

**Fig. 1:**
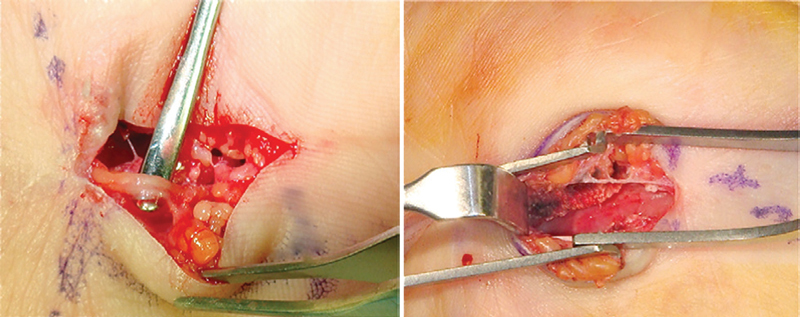
Mini-open carpal tunnel release.

The beginning of development of these techniques is marked with creating minimally invasive techniques in the surgery of compressive neuropathies. Classical open technique for carpal tunnel decompression involved V-shaped or curvilinear skin incision from the middle of the palm and proximally along the first several centimeters of forearm to expose median nerve. Minimally invasive incision is only 2 to 3 cm in the middle of the palm until the first carpal crease ( Fig. 1 ). This incision enables the surgeon to visualize whole flexor retinaculum, and to safely cut it without damaging recurrent motor branch. Besides better cosmetic effect, there is less damage to vascularization of the nerve that sometimes occurred during unnecessary exposure of median nerve in the forearm. 8

After this, more and more minimally invasive approaches for peripheral nerve injuries were published in the literature. Since the number of these techniques is too large, we will mention only few in this review.

**Fig. 2:**
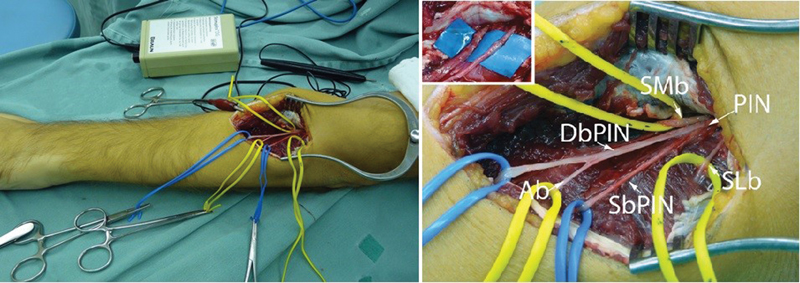
Photograph of the incision used to transfer supinator branches to the posterior interosseous nerve in the left upper limb. Medial (SMb) and lateral (SLb) branches, and the posterior interosseous nerve (PIN). The particularity in this patient was a proximal division of the PIN into its superficial (SbPIN) and deep (DbPIN) branches. The superficial posterior interosseous innervates the extensor digitorum communis, the extensor digit quinti, and the extensor carpi ulnaris. The deep branch of the posterior interosseous nerve innervates the abductor pollicis longus (Ab), the extensor pollicis longus, and the extensor indicis proprius. 18

One of the examples is the nerve transfer of supinator motor branch to the posterior interosseous nerve. 18 The incision is made from lateral epicondyle until distal third of forearm, between extensor carpi radialis muscle and extensor digitorum muscle. After separation of these two muscles, you can clearly see supinator muscle. Posterior interosseous nerve is then found by palpation and after that branches for supinator muscles are identified and dissected ( Fig. 2 ).

In brachial plexus lesions, to enable extension of the wrist, nerve transfer of motor branch of the pronator quadratus muscle and motor branch of extensor carpi radialis brevis muscle can be used. Although previously considered very difficult to treat, and can be found in some literature that there is poor recovery after distal transfers, recent literature showed that repair of these nerve elements is followed by good recovery. 19 In this approach, the incision is in the middle of the forearm, 8 cm proximally from the carpal groove. Median nerve is found between the flexor digitorum communis muscle and the flexor pollicis longus muscle. The dissection is then continued to the interosseous membrane of the forearm where the anterior interosseous nerve can be found. Following this, the nerve pronator teres is found. Branches of flexor pollicis longus can be found and they are dissected from the anterior interosseous nerve. Motor branch can be found next to radial nerve, using additional incision. Then deeper and next to it, motor branch of extensor carpi radialis brevis can be found. Section of this branch is made and flipped distally, while proximal end of anterior interosseous nerve is flipped proximally and transpositioned under pronator teres muscle. With this, the nerve transfer can easily be done.

**Fig. 3:**
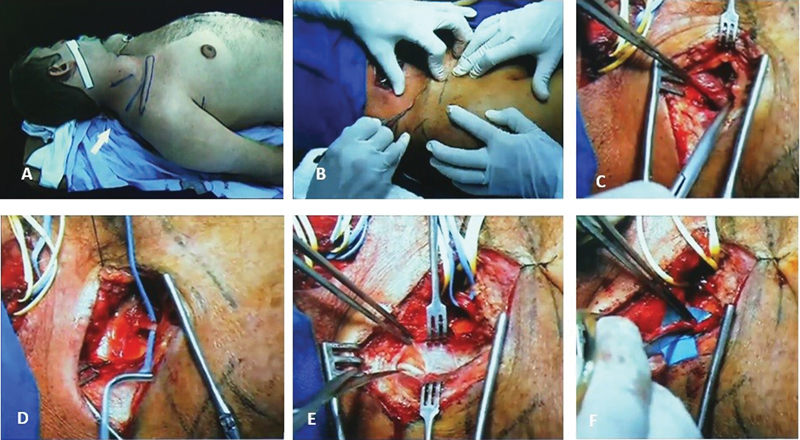
Nerve transfer of accessory nerve to the suprascapular nerve. 20 ( **A** ) Patient positioning and skin incision. ( **B** ) Beginning of the surgery. ( **C** ) Dissection of omohyoid muscle. ( **D** ) Dissection of suprascapular nerve. ( **E** ) Dissection of accessory nerve on anterior margin of trapezius muscle. ( **F** ) Nerve stumps prepared for nerve transfer.

Not only peripheral nerves can be treated using minimally invasive approaches but also brachial plexus lesions. One of these approaches is created for nerve transfer of accessory nerve to suprascapular nerve. 20 Incision is made in length of 4 cm at approximately 90-degree angle in reference to clavicle ( Fig. 3 ). After dissection of soft tissue, omohyoid muscle is visualized and suprascapular nerve is found just beneath this muscle. After this, anterior margin of trapezius muscle is palpated and its fascia is then incised. At the place where this muscle meets, deep cervical fascia accessory nerve and its branches can be found. After sectioning of these nerves and preparation of both nerve stumps with just a few epineural stitches, nerve transfer is completed.

### Minimally Invasive Endoscopic Peripheral Nerve and Brachial Plexus Surgery

**Fig. 4:**
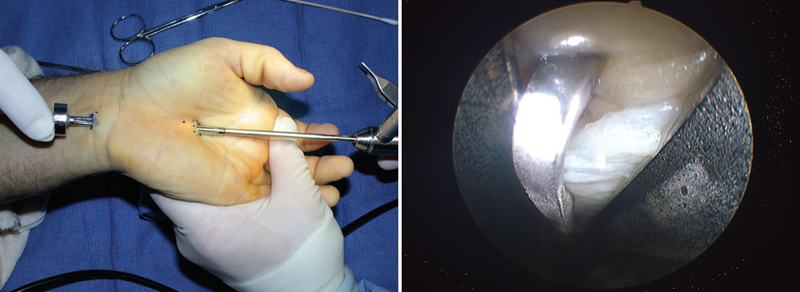
Endoscopic carpal tunnel release—biportal technique. 21

Endoscopic carpal tunnel release (ECTR) has been performed since the late 1980s, using two operating techniques. Advantages of ECTR are shorter recovery time, less postoperative pain, reduced postoperative wound sensitivity, and less scaring. Disadvantages are steep learning curve; less visibility, which may result in incomplete sectioning of the TCL and increased neurovascular injury; and increased cost associated with endoscopic instruments. Several published studies showed excellent results using this technique. Hankins et al showed 82.6% of complete recovery using Brown's biportal technique ( Fig. 4 ) 21 , while Chen et al had 91% of complete recovery using Menon's uniportal technique. 21 22 There were also attempts to treating cubital tunnel syndrome using endoscopy. Tsai et al reported 64% success in their series of 85 cubital tunnel releases. 23 Ahcan and Zorman showed even better results—in their series, good or excellent result was achieved in 91% of patients. 24 While only “in situ” decompression was performed in these series, decompression was followed by subcutaneous transposition in the study by Krishnan et al of 11 treated patients, with excellent results in 63.7%, good in 27.3%, and satisfactory in 9.1% patients. 25

**Fig. 5:**
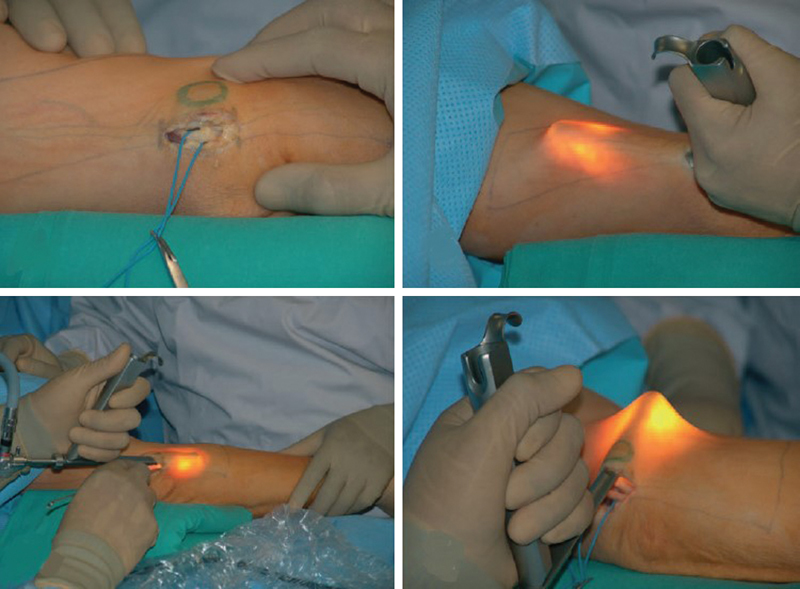
Endoscopic cubital tunnel release. 25

Attempts at endoscopic cubital tunnel release are also reported. Zlowodzki reported four randomized controlled trials. Two of these studies used the submuscular transposition and the other two used the subcutaneous technique. A total of 261 patients were involved in the study with an average follow-up of 21 months. Complication rate was 9% for the group of patients treated with “in situ” decompression and 30% in the group treated using anterior subcutaneous transposition technique. 26 Tsai et al reported 85 endoscopic cubital tunnel releases through a 2- to 3-cm incision over the course of the ulnar nerve (UN) at the elbow, and the authors were able to decompress up to 10 cm proximal and 10 cm distal to the medial epicondyle. In this series, 64% showed improvement after surgery, but two patients subsequently required transposition procedures for recurrent symptoms. 23 Ahcan and Zorman reported endoscopic release of a 20-cm segment of UN via a 3.5-cm incision overlying the cubital tunnel with good or excellent results achieved in 91% of patients ( Fig. 5 ). 24

**Fig. 6:**
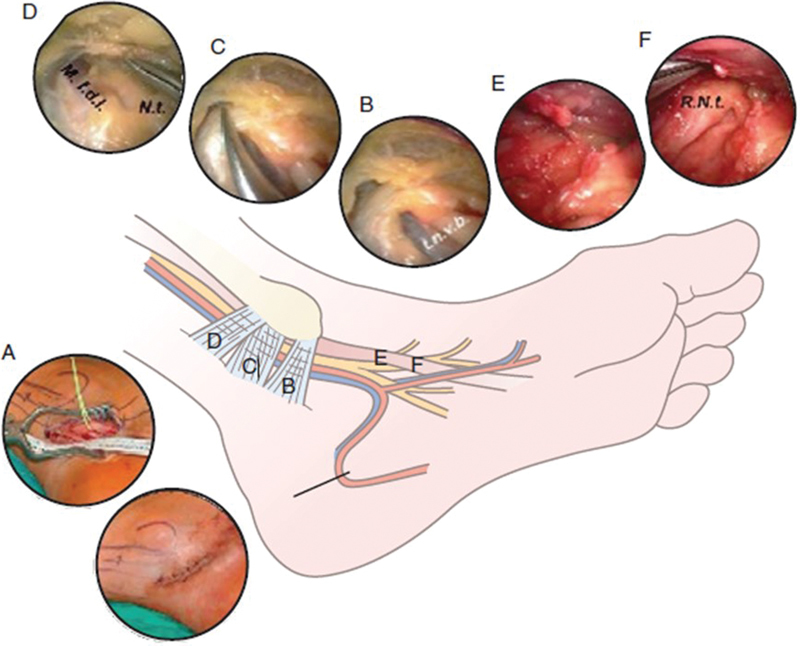
Endoscopic tarsal tunnel release. 25

Surgery for tarsal tunnel syndrome can also be performed using minimally invasive endoscopic approach with promising results—82% had excellent recovery in Mullick and Dellon's series of 87 treated patients ( Fig. 6 ). 26

Endoscopic surgery for brachial plexus is still in the development stage. Even though the technology has made huge leap in the past years, sometimes exact localization and type of lesion cannot be established, and so open surgical exploration is necessary. A few cadaveric trials using surgical robotic systems were conducted in attempt to find a minimally invasive technique for exploration of the brachial plexus, which would also be possible to make surgical reparation of the injured nerve. 27

**Fig. 7:**
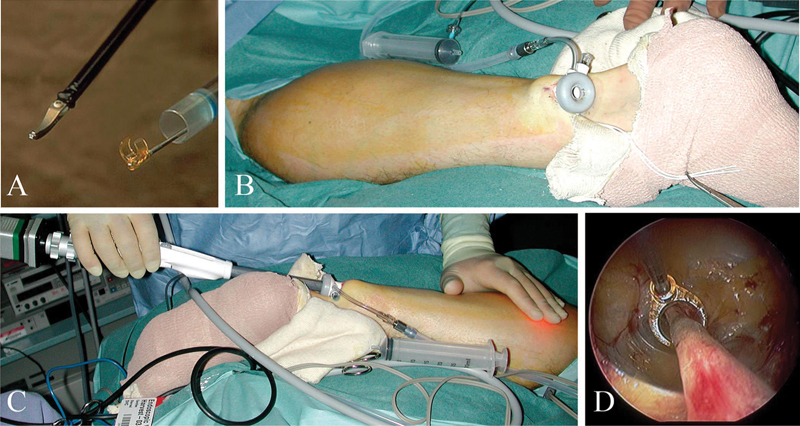
Endoscopic extraction of sural nerve using bipolar dissector and C-ring with retraction system Guidant VasoView Uniport Plus. 28

Another interesting application of endoscope in peripheral nerve surgery is in sural nerve harvesting. As we know, sural nerve is probably the most frequently used donor for nerve grafting. Usual open approach for sural nerve harvesting is done by making a series of small incisions in the path of this nerve. In the past few years, a new method was developed—endoscopic sural nerve harvesting. Duration of the procedure is approximately 25 minutes and requires only one skin incision of length of 12 mm, instead three incisions used in classical open approach ( Fig. 7 ). 28

## Conclusion

Together with technological progress, peripheral nerve and brachial plexus surgery made its improvements. Use of microsurgical technique, operative microscope, and modern materials made huge difference in the treatment outcome of peripheral nerve and brachial plexus surgery. In some cases, it is impossible to use minimally invasive approach due to characteristics of the lesion, but with further technological advances, more and more cases each year can be safely treated either with minimally invasive open surgical approaches or endoscopic approaches. Using minimally invasive treatment, trauma of the tissue is less, the incision is smaller, and there is less scarring; however, chances for iatrogenic lesion of nerve and vascular elements is higher.

Improvement in presurgical evaluation leads to more precise determination of type and location of the injury, decreasing the need for complete exploration of the peripheral nerve and brachial plexus and enabling usage of smaller incisions—specific approaches for specific types of lesions. Multidisciplinary approach individually designed for every patient is of the utmost importance for successful treatment of peripheral nerve and BPIs. In the future, integration of biology and nanotechnology may fabricate a new generation of nerve conduits that will allow nerve regeneration over longer nerve gaps and start new chapter in peripheral nerve surgery.
